# ‘I wanted to safeguard the baby’: a qualitative study to understand the experiences of Option B+ for pregnant women and the potential implications for ‘test-and-treat’ in four sub-Saharan African settings

**DOI:** 10.1136/sextrans-2016-052972

**Published:** 2017-07-23

**Authors:** Estelle McLean, Jenny Renju, Joyce Wamoyi, Dominic Bukenya, William Ddaaki, Kathryn Church, Basia Zaba, Alison Wringe

**Affiliations:** 1 Malawi Epidemiology and Intervention Research Unit, Karonga, Malawi; 2 Faculty of Epidemiology and Population Health, London School of Hygiene and Tropical Medicine, London, UK; 3 National Institute for Medical Research, Mwanza, Tanzania; 4 Medical Research Council/Uganda Virus Research Institute Research Unit on AIDS, Entebbe, Uganda.; 5 Rakai Health Sciences Program, Kalisizo, Uganda

**Keywords:** ANTERETROVIRAL THERAPY, AFRICA, HIV, PREGNANCY, QUALITATIVE RESEARCH

## Abstract

**Objective:**

To explore what influences on engagement with Option B+ in four sub-Saharan African settings.

**Methods:**

In-depth interviews were conducted in 2015, with 22 HIV-positive women who had been pregnant since Option B+ was available, and 15 healthcare workers (HCWs) involved in HIV service delivery. Participants were purposely selected from four health and demographic surveillance sites in Malawi, Tanzania and Uganda. A thematic content analysis was conducted to investigate what influenced engagement with Option B+.

**Results:**

Feeling ‘ready’ was key to pregnant women accepting antiretroviral treatment (ART) on the same day as diagnosis at antenatal clinic; this was influenced by previous knowledge of HIV-positive status, interactions with HCWs and relationship with their partners. The desire to protect their unborn infant was the main issue that motivated women to initiate treatment, temporarily over-riding barriers to starting ART. Many HCWs recognised that pressurising women into starting ART may lead them to stop treatment following delivery. However, their own responsibility to protect the infant sometimes drove HCWs to use strong persuasive techniques to initiate pregnant women onto ART as early as possible, occasionally causing women to disengage.

**Conclusions:**

Protecting the baby superseded feelings of unpreparedness for lifelong ART and may explain poor retention observed in Option B+ programmes. Women may benefit from more time to accept their status, and counselling on the long-term value of ART beyond the pregnancy and breastfeeding period. Strategies to promote readiness for same-day initiation of lifelong treatment are urgently needed, and may provide important lessons for universal test-and-treat implementation.

## Background

Initiating pregnant and breastfeeding women on lifelong antiretroviral therapy (ART) regardless of clinical stage or CD4 count (Option B+) was expected to ease the burden on health services by removing the need to manage ART interruptions, and to protect women's partners and future pregnancies by reducing viral rebounds.^1^ Option B+ was devised and implemented by Malawi in 2011; it was incorporated into WHO guidelines in 2012^2^ and implemented by several other African countries, including Uganda and Tanzania, in 2013.^3^

Programmatic evaluations of Option B+ in many African settings have indicated higher rates of enrolment,^3–5^ but higher levels of attrition within the first year, leading to poorer retention in care compared with programmes for women who start ART for their own health.^6–8^ Low retention presents challenges for meeting the UNAIDS ‘90–90–90’ targets^9^ and may lead to drug resistance, increasing the risks of treatment failure if women reinitiate ART later.

Qualitative studies exploring social and contextual factors surrounding the acceptance and retention of Option B+ have identified fear of involuntary disclosure of HIV status, difficulties with clinic staff, transportation issues and lack of support from partners as key barriers.^10–13^ These issues resonate with findings from studies exploring engagement with HIV services among non-pregnant people living with HIV (PLHIV).^14^ It is therefore not apparent why patterns of uptake and adherence differ among women who initiate ART under Option B+ compared with women in standard ART programmes. Furthermore, few studies have explicitly explored how psychosocial issues relating to pregnancy interplay with these factors.

‘Test-and-treat’ policies are being rolled out in many African countries,^15^ which will involve ART-initiation for all PLHIV immediately following an HIV-diagnosis regardless of their immunological status, many of whom will not have had AIDS symptoms. As there are parallels between these asymptomatic PLHIV and pregnant women being asked to accept Option B+, it may be possible to use these women's experiences to help guide implementation of ‘test-and-treat’.

In this paper, we aim to understand what influences acceptance and adherence to Option B+, and explore whether these influences are specific to pregnancy, using data from a multicountry qualitative study in rural areas of Malawi, Tanzania and Uganda.

### Theoretical perspectives

**Figure 1:**
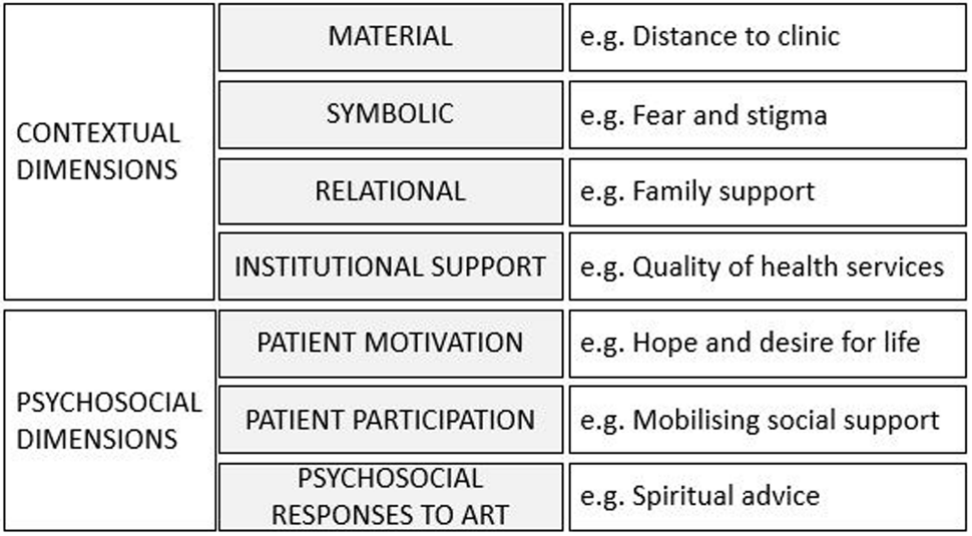
Skovdal *et al*'s expanded socioecological framework (adapted from Ref. 17).

A socioecological model^16^ extended to include additional psychosocial dimensions (figure 1) has previously been used to explore factors relating to uptake and retention on ART in PLHIV.^17^ In our analysis, we draw from this framework to disentangle the specific issues relating to pregnancy and medication-taking when living without symptoms of HIV-related illnesses.

## Methods

Data for this analysis were drawn from the Bottlenecks Study undertaken in seven health and demographic surveillance sites (HDSS) across six countries in Eastern and Southern Africa within the ALPHA network (http://alpha.lshtm.ac.uk/). The aim of the broader study was to understand how differences in HIV policy and programme implementation influence the healthcare-seeking experiences of PLHIV in Eastern and Southern Africa.

### Study settings

This paper presents a subsection of the Bottlenecks data from settings where Option B+ women were interviewed: Karonga (Malawi), Kisesa (Tanzania), Kyamulibwa and Rakai (Uganda), where HIV prevalence since ART was available was 7.4%, 5.6%, 6.6% and 12.6%, respectively.^18^ Crude fertility rates per 1000 since ART was available were 158.3 in Kisesa, 149.7 in Kyamulibwa^19^ and 166.1 in Karonga (personal communication). Option B+ was available in Karonga in 2012, in Kyamulibwa and Rakai in 2013, and in Kisesa in 2014. All four study locations are rural, with most residents involved in subsistence farming.

### Sampling frame and participants

A sampling frame of PLHIV was drawn up in each setting using HDSS records, clinic data and screening tools. Participants were then purposively selected to include those who had not yet initiated ART, had been on ART for various lengths of time or were lost to follow-up. For this substudy, all 22 female participants who were pregnant, or had been since Option B+ had been available, were included: nine from Malawi, five from Tanzania and nine from Uganda. The median age of these participants was 30, with a median of three live births, 16 were in relationships and two reported taking ART previously. Healthcare workers (HCWs) involved in providing HIV care at local clinics were purposively sampled; 15 were included in this analysis. There were no refusals to participate; however, one interview in Malawi was cut short when the woman did not want to continue. Further information can be found in the online supplementary file found in the editorial at http://dx.doi.org/10.1136/sextrans-2017-053172.^20^

### Data collection

Due to the sensitive and personal nature of information to be discussed, face-to-face in-depth interviews (IDIs) were used. The IDIs were conducted in 2015, by experienced research assistants in the local languages (Swahili in Kisesa, Tumbuka in Karonga, Luganda in Rakai and Kyamulibwa). IDIs lasted 60–90 min on average, and were conducted in private at participants' homes or in the clinics. Topic guides covered social and economic circumstances and experiences of using or providing HIV care and treatment services, including during pregnancy. Field notes were taken and IDIs were audio-recorded, and were translated to English verbatim (Karonga, Kisesa, Rakai) or summarised into detailed reports (Kyamulibwa) by research assistants. Transcripts/reports were anonymised, and all data stored in secure password-protected locations. Participants were recruited until data saturation was reached; IDIs were repeated occasionally to explore certain topics in more detail. Additional methodological details concerning the Bottlenecks study can be found in an online-only supplement.

### Analysis

An initial thematic content analysis was conducted by each local study coordinator who coded the data, assisted by NVivo 10 or 11 in Karonga, Kisesa and Rakai and conducted manually in Kyamulibwa. Emerging themes based on experiences, opinions and understanding of Option B+ were summarised in tables. Summary tables from each of the four settings were compiled, and raw transcripts/summaries were used to expand on relevant thematic areas. The emerging themes were compared with the expanded socioeconomic framework (figure 1); relevant elements of this framework plus additional important themes were then used to create a framework specific to this population.

### Ethics

This study was approved by ethical review boards in each country and at the London School of Hygiene and Tropical Medicine.

## Results

**Figure 2:**
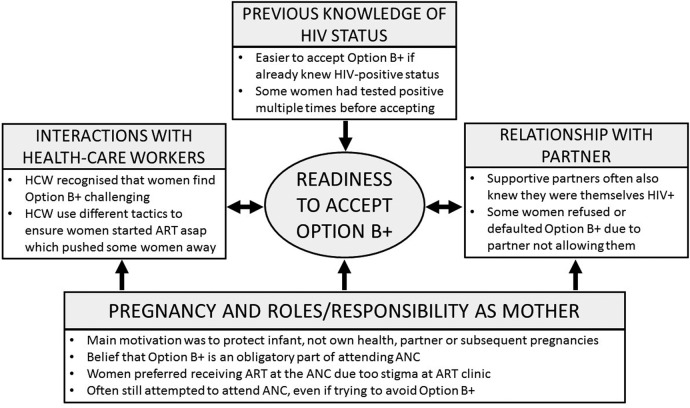
Framework of factors influencing women's readiness to accept Option B+. ART, antiretroviral treatment; ANC, antenatal clinic; HCW, healthcare workers.

Several inter-related themes that went beyond the extended socioeconomic model emerged from our analysis to explain engagement with HIV treatment among pregnant women in the four settings (figure 2).

### The role of pregnancy in motivating ART initiation

For most participants, the prevailing motivation driving uptake and adherence to Option B+ was to protect the unborn child from HIV, and to stay alive to support the child after birth.

I felt uncomfortable [following HIV diagnosis] but they told me that I should not feel bad about it. They assured me that I would be able to have a child without HIV. (PLHIV––accepted B+ but defaulted after birth)

I wanted to start on the treatment earlier to deliver a baby free of HIV. (PLHIV––accepted B+ and retained)

HCWs recognised this driving force to protect the child, and some expressed concerns that women would discontinue taking ART once they had weaned the baby.

… when they get to know the HIV negative status of their children, they do not go back for more treatment and care: they only think about the health of the children and forget about theirs. (HCW)

No patient or HCW mentioned protecting subsequent pregnancies, and the health of the woman was rarely mentioned. Few noted the additional benefit of Option B+ in reducing risk of transmission to HIV-negative partners.

### The readiness of women to accept Option B+

Women who first tested positive at the antenatal clinic (ANC) often found it difficult to accept starting ART. This was recognised by HCWs, who also suggested that those who were young, in their first pregnancy, or lacking support were the most vulnerable, as in this example of a 15-year-old girl:

…we diagnosed her with HIV and we advised her to start taking ARVs, but that girl refused and tore her health passport because it was an unexpected thing to her…. (HCW)

Conversely, women who found it easier to accept the need for ART had often already come to terms with their positive status, like this woman who had tested multiple times in different places before believing the result, reporting that ‘…I just felt that I am ready to start taking them [ART] because…I took the test, in all the places for about four times…I just realized that I have to take them’ (PLHIV––accepted B+ and retained).

In each country, policy states that pregnant women can choose to opt out of HIV testing and treatment. However, most women believed that HIV testing and ART initiation were obligatory parts of antenatal clinic.

The nurse tested my blood as it is an instruction of the hospitals to test pregnant women. She found that I had a sickness [HIV] which deserved that treatment, so I had no alternative…I accepted to take the drugs, because I had no option. (PLHIV––accepted B+ and retained)

The majority of HCWs recognised that receiving an HIV diagnosis and having to start ART immediately ‘is not an easy situation…she can't just receive it abruptly’ (HCW). HCWs reported that in general women went through the counselling processes with no issues; however, many also felt that ‘we need to give people time to take a decision. We may force them to begin HIV treatment and they refuse to take the medicine as prescribed’ (HCW). Despite these sentiments, most HCWs felt bound by the policy to start all eligible women on ART on the same day:

…we ask that person the reasons she doesn't want to test once she has explained to me the reasons, I refer that person to my friend since we have different ways of handling people you may find that she accept to be tested. If they are not on ART we counsel them and tell them the reasons why we are doing that up until they accept…From there we also go together to the clinic I always make sure she gets there. They don't go alone [to start ART] because if you can send them alone sometimes they cannot go. (HCW)

If they decline HIV testing, we tell them that they cannot get treatment from our facility. (HCW)

Pregnant women who did not feel ready to initiate ART described various avoidance tactics, such as accepting then discarding treatment, providing false information so that they could not be followed up or switching ANC by obtaining new health documents. These behaviours were well known to HCWs in all settings.

Yes I was tested [at first ANC] and found positive again and I was told to go to [hospital] to start ART because at [first ANC] there were no ARVs…I didn't go I then went to [second ANC] because I had backache they asked me if I already started ANC I said no … I then tested positive and was told to start ART. At [second ANC] I went with another Health passport not the one I was using at [first ANC]. (PLHIV––accepted B+, but defaulted while still pregnant)

### The effect of interpersonal interactions on acceptance of Option B+

Women's relationships with the HCWs were generally reported to be good by both women and HCWs. However, the accounts of women illustrated a clear power hierarchy and little two-way communication. While many women accepted what they were told without question, some complained reporting they ‘were not taught anything’ (PLHIV*––*did not accept B+) or ‘did not get any counselling’ (PLHIV––accepted B+, still pregnant or breast feeding), or that the HCWs shouted at them if they missed appointments. These interactions served as drivers to some who felt obliged to follow the ‘rules’ but as barriers to others who opted to disengage with care completely.

Additionally perceived or actual support from male partners emerged as a factor in determining whether a woman accepted Option B+ or not. When a woman's husband was supportive, he was often also HIV-positive (or suspected it). Some women with supportive partners reported themselves as the lucky ones as ‘some men are abusive but my husband does not do that’ (PLHIV––accepted B+ but defaulted after birth). In all settings, there were examples of unsupportive partners. Some pregnant women were not taking ART due to their husband disallowing them, as explained by this woman:

…there were quarrels with my husband in my house: he said ‘I don't know about HIV’, ie, why I have stayed without going to hospital until now (PLHIV––did not accept B+).

## Discussion

Our qualitative study in four rural African settings found that the psychosocial factor of feeling ‘ready’ was key to being able to accept ART on the same day as diagnosis at ANC. This was influenced by many factors, including previous knowledge of their HIV-positive status, interactions with HCWs and the relationship with their partner. Underpinning all of these remained the driving influence of the pregnancy and role as a mother (figure 2). Pregnancy may result in women accepting ART before they feel ‘ready’, and then stopping treatment once its influence has passed. The limitations of the extended socioecological model among this population illustrate the potential differences between pregnant women and non-pregnant PLHIV. This difference could explain the high level of loss to follow-up in the first few months after uptake of Option B+ observed in recent quantitative studies.^8^

The importance of women feeling ready to start ART was mentioned by HCWs and women in all settings. Accepting a positive result took time, and often several ‘confirmatory’ tests over months and years.^21^ Like others, our study suggests that those who were newly diagnosed or more vulnerable, such as those who were younger or had an unsupportive partner, were more likely to struggle with accepting ART.^22–25^ In all the settings, women seemed to go along with what the HCWs said even if they did not agree with it, including thinking that HIV testing in the ANC was obligatory. This has also been found in several other countries in Africa, including Malawi, Tanzania and Uganda.^26–28^

The woman's role as a mother was a powerful motivator to accept ART even if they did not feel entirely ‘ready’, or to continue to attend the ANC despite wanting to avoid HIV testing or ART initiation. For many women, it appears that the will to protect the infant drove them to overcome or ignore other socioecological barriers such as poor support, provider attitudes, health facilities issues and side effects (data not presented) to be able to continue taking ART. Like others, we found that HCWs presented the protection of the infant as the main objective of Option B+,^25^ perhaps enabling them to justify their adoption of strong persuasive techniques. Young age and vulnerability due to the pregnancy may have contributed to women's willingness to obey the HCWs. The subservience illustrated by the women's willingness to obey HCWs without question could be attributed to imbalances in age, education and social status between the patients and providers, particularly within these social, cultural and political contexts.^28^ ^29^

Once the woman has fulfilled her objective of protecting the baby, not only has her main motivation to take ART reduced, but also her reason to attend a clinic and obey the HCWs. The support she received from her partner to attend may diminish, and she might experience further stigma while attending the general ART clinic that she did not experience at the ANC. The concern that women will drop out of care after delivering an HIV-negative baby has been expressed in many settings.^11^ ^12^ ^30^ We found that HCWs were aware that pressurising women to accept Option B+ before they were ready might cause them to default from treatment; however, they were constrained from giving women more time due to a desire to protect the baby as soon as possible. Asymptomatic male and non-pregnant female PLHIV targeted by future ‘test-and-treat’ policies may also struggle to accept lifelong ART immediately, especially without a strong time-bound motivator like pregnancy. The absence of this window to protect the infant may mean that HCWs also use less pressure, which may have implications for treatment engagement in either direction.

There are various strengths and limitations to our study. As it was nested within a larger study with a broader focus, it was not possible to explore all topics relating to Option B+ in the interviews, including, for example, women's and HCWs’ views on how to improve the services. There was a potential for social desirability bias in respondents' accounts (eg, wanting to appear as a ‘good mother’ or a ‘good patient’); however, research assistants were given extensive training with a specific focus on developing rapport and trust with the participants to reduce this. Although HDSS populations may differ from the general population, a key strength of the study was our ability to draw on data from four areas: we found striking similarities in the issues that emerged across the different settings, making it likely that our findings can be generalised to pregnant women in other rural populations in sub-Saharan Africa, though urban populations may be different. Furthermore, through our use of the HDSS datasets, we could identify women who had refused Option B+ or had stopped taking ART, who are often omitted from studies that rely on sampling frameworks from clinic registers. However, these women tended to be difficult to interview: despite efforts to reassure them, some women displayed similar avoidance tactics as they might use to avoid taking the ART. Due to the need to disclose their HIV status to the interviewer, women who had not accepted their positive HIV status would have been missed by this study; it would also be important to understand why such women totally disengage from care.

## Conclusions

We have shown that being pregnant and one's role as a mother was an overwhelming, yet time-limited influence on acceptance and adherence to Option B+ in these settings in Malawi, Tanzania and Uganda. To combat the higher attrition rates in pregnant women starting ART due to Option B+, women may benefit from being given more time to accept their status and receiving counselling tailored to their specific needs with a focus on more sustainable reasons for taking the ART, such as their own health and protecting future pregnancies, especially in high-fertility areas. Other PLHIV may have a similar range of factors influencing their readiness to start ART immediately following a positive HIV test. The absence of such a strong motivator to protect the unborn child and the link to specific tailored ANC services may further hamper abilities to overcome these barriers: this should be borne in mind when implementing ‘test-and-treat’ policies.

**Key messages**

- Readiness to start antiretroviral treatment (ART) while pregnant depended on multiple factors; however, the drive to protect the unborn child enabled many women to temporarily overcome all other barriers.
- Without this motivation, newly diagnosed patients expected to start ART immediately through ‘test-and-treat’ programmes may find barriers to starting treatment more difficult to overcome.
- Healthcare workers were aware of the challenges of accepting Option B+, but their desire to protect the infants and comply with policy obligated them to adopt strong persuasive tactics.
